# Action of YM155 on clear cell renal cell carcinoma does not depend on survivin expression levels

**DOI:** 10.1371/journal.pone.0178168

**Published:** 2017-06-05

**Authors:** Mei Yi Sim, Hung Huynh, Mei Lin Go, John Shyi Peng Yuen

**Affiliations:** 1 Department of Urology, Singapore General Hospital, Republic of Singapore; 2 Laboratory of Molecular Endocrinology, Division of Molecular and Cellular Research, National Cancer Centre, Republic of Singapore; 3 Department of Pharmacy, National University of Singapore, Republic of Singapore; Medical College of Wisconsin, UNITED STATES

## Abstract

The dioxonapthoimidazolium YM155 is a survivin suppressant which has been investigated as an anticancer agent in clinical trials. Here, we investigated its growth inhibitory properties on a panel of immortalized and patient derived renal cell carcinoma (RCC) cell lines which were either deficient in the tumour suppressor von Hippel-Lindau (VHL) protein or possessed a functional copy. Neither the VHL status nor the survivin expression levels of these cell lines influenced their susceptibility to growth inhibition by YM155. Of the various RCC lines, the papillary subtype was more resistant to YM155, suggesting that the therapeutic efficacy of YM155 may be restricted to clear cell subtypes. YM155 was equally potent in cells (RCC786.0) in which survivin expression had been stably silenced or overexpressed, implicating a limited reliance on survivin in the mode of action of YM155. A follow-up *in-vitro* high throughput RNA microarray identified possible targets of YM155 apart from survivin. Selected genes (*ID1*, *FOXO1*, *CYLD*) that were differentially expressed in YM155-sensitive RCC cells and relevant to RCC pathology were validated with real-time PCR and western immunoblotting analyses. Thus, there is corroboratory evidence that the growth inhibitory activity of YM155 in RCC cell lines is not exclusively mediated by its suppression of survivin. In view of the growing importance of combination therapy in oncology, we showed that a combination of YM155 and sorafenib at ½ x IC_50_ concentrations was synergistic on RCC786.0 cells. However, when tested intraperitoneally on a murine xenograft model derived from a nephrectomised patient with clear cell RCC, a combination of suboptimal doses of both drugs failed to arrest tumour progression. The absence of synergy *in vivo* highlighted the need to further optimize the dosing schedules of YM155 and sorafenib, as well as their routes of administration. It also implied that the expression of other oncogenic proteins which YM155 may target is either low or absent in this clear cell RCC.

## Introduction

Renal cell carcinoma (RCC) is a lethal form of genitourinary cancer that is notoriously resistant to traditional cytotoxic chemotherapy and radiotherapy [1]. Of the various histological subtypes, the clear cell variant is the most prevalent, accounting for 75–80% of reported cases. Clear cell RCC is either sporadic (>96%) or familial (< 4%) [2,3]. The pathology of clear cell RCC is critically dependent on the tumour suppressor von Hippel-Lindau gene (*VHL*), whose protein product regulates cellular responses to hypoxia [4]. Inactivation of *VHL* is specific to clear cell RCC and not observed in other histological cell types such as papillary, chromophobe and collecting duct RCCs [1].

Survivin, the smallest member of the Inhibitor of Apoptosis (IAP) protein family [5,6], is selectively overexpressed in almost every human tumour [7,8,9,10] and consistently identified as a risk factor for poor prognosis and disease recurrence. In malignant tissues, survivin expression is linked to suppression of apoptosis, metastasis, by-pass of cell cycle checkpoints and resistance to chemotherapy [11,12,13]. Various strategies have been employed to suppress survivin activity such as antisense oligonucleotides, small molecule suppressants and survivin-based vaccination [14]. Among small molecules, the dioxonaphthoimidazolium analog YM155 has been extensively investigated [15,16,17]. YM155 blocks the transcription of the survivin gene (*BIRC5*) [18,19] and induces dissociation of the ILF3-p54nrb complex which positively regulates survivin expression [20]. There is a growing body of evidence that the anti-cancer properties of YM155 does not arise solely from the inhibition of survivin [17]. The involvement of other oncogenic proteins (MCL1, PI3K, ERK, STAT3, securin) [21,22,23] and signaling pathways (NF-κB, autophagy) [24,25] have been implicated in its mode of action. Though YM155 is not a specific survivin suppressant, it should not detract from the fact that it is a potent and well tolerated anti-cancer candidate [26,27,28], with the potential to enhance the efficacies of established anticancer drugs when used in combination. We hypothesized that YM155 intercepts other molecular targets apart from survivin to bring about growth inhibition of RCC.

In this study, we aim to investigate the mechanism of action of YM155 in the inhibition of survivin and RCC.

## Materials and methods

### Cell culture

RCC786.0, ACHN and A498 were obtained from American Type Culture Collection (ATCC, Manassas, VA, USA). Two human clear cell RCC cell lines, 786.0/EV and 786.0/VHL were obtained from Cancer Research UK Laboratories, Clare Hall, Hertfordshire, UK. Three patient-derived RCC cell lines (NCC010, NCC035, P.RCC) were obtained from NCCS-VARI Translational Research Laboratory, National Cancer Centre Singapore. All cells were cultured in high glucose Dulbecco’s Modified Eagle Medium (DMEM) containing L-glutamine, sodium pyruvate, 10% FBS and 100 U Penicillin/Streptomycin (Gibco, Waltham, MA, USA) and grown in a humidified atmosphere of 5% CO_2_-95% filtered atmospheric air maintained at 37°C. RCC786.0/EV and RCC786.0/VHL.HA were stably transfected with empty vector or HA-tagged wild type *VHL* respectively.

### Patient-derived RCC xenograft in SCID mice

Clinical specimens were obtained from RCC patients who had undergone nephrectomy. Sample collection was carried out with written informed consent from patients and approval from the Institution Review Board of the Singapore General Hospital. All written consent were filed and kept under lock and key to ensure patient confidentiality. Specimens from nephrectomy were obtained intra-operatively. The diagnoses of RCC were confirmed by histology for all cases. The experiments were carried out on mice that were homozygous for the SCID mutation [29], with approval from the hospital’s Institutional Animal Care and Use Committee and based on guidelines described for the welfare and use of animals in cancer research [30].

As described previously [31], freshly sectioned RCC tissues were placed in RPMI 1640 in an ice bath immediately on tumour sectioning. Thin slices of the tumour tissue, taken during the preparation of slices for cryostat sections but before processing into permanent paraffin-embedded sections, were weighed, diced into 2–3mm^3^ pieces, and washed three times with RPMI 1640 medium. They were minced finely to give tissue fragments that could pass through an 18-gauge needle, then mixed 1:1 (v/v) with Matrigel to give a total volume of 0.2mL per injection which was then administered subcutaneously (SC) to the right flank of a 8–10 week-old male SCID mouse. This was repeated on 4 other mice. Mice were monitored for general well-being and tumour size was measured at least twice weekly for 5 months. For serial transplantation, tumour-bearing animals were killed by CO_2_ exposure. Animals were placed in an ice water bath (2 min), dipped in and out of 10% Clorox solution for 2 min, washed in four changes of ice water, placed in 70% ethanol, and transferred to a laminar flow hood for dissection. Tumours were minced under sterile conditions and tissue fragments that passed through an 18-gauge needle were mixed with Matrigel for serial transplantation to successive SCID mice.

Treatment with YM155 (1-(2-methoxyethyl)-2-methyl-4,9-dioxo-3-(pyrazin-2-ylmethyl)-4,9-dihydro-1H-naphtho[2,3-d]imidazolium bromide, Shanghai BioChem Partner, People’s Republic of China, 98% purity) was initiated when the average tumour size was approximately 200 mm^3^ (about two weeks post implantation). Briefly, xenograft-bearing mice (10 per group) were administered (intra-peritoneal) with vehicle (PBS), or YM155 at doses of 1, 2 or 3 mg/kg (2x daily 15 days), or orally administered 200 μl of vehicle (30% Capsitol in water), or sorafenib tosylate at a dose of 10 mg/kg (1x daily 15 days), or a combination treatment with 2 mg/kg YM155 and 10 mg/kg sorafenib. Growth of established xenografts was monitored at least twice weekly by measuring the tumour length (a) and breadth (b) with Vernier calipers to give tumour volume (= [a−b^2^] /2). Animals were sacrificed 12h after the last treatment dose, body and tumour weights were recorded and tumours were snapped frozen in liquid nitrogen and stored at -80°C for further analyses.

### Transient transfection with siRNA duplexes

siRNA duplexes (S1 Table) were supplied by Qiagen (Hilden, Germany). RCC786.0/EV, RCC786.0/HA.VHL and NCC010 were transfected with survivin siRNAs using Oligofectamine^®^ (Invitrogen, Waltham, MA, USA) according to manufacturer’s instructions. Briefly, cells were seeded at a density of 30% confluence for transfection and transfected cells were grown at 37°C up to day 13 prior to further manipulation. Proprietary negative control siRNAs (Qiagen, Hilden, Germany) with no known homology to any mammalian gene were used as the negative control.

### Clonogenic survival assay

To assess the ability of the transfected cells to survive and replicate, clonogenic survival assays were performed as described [32]. Forty-eight hours after siRNA transfection, cells were disaggregated and seeded in 10-cm dishes (in triplicates) at 1000–1500 cells/dish. The cells were incubated at 37°C, 5% CO_2_ for 10–14 days until discreet colonies were observed. Cell colonies were fixed in methanol:acetic acid (3:1) and stained with Giemsa Methylene Blue (Merck). Visible colonies were manually counted. The remaining transfected cells were stored to assess survivin protein levels.

### Stable transfection and selection of survivin knockdown or over-expressed cells

Transfection of RCC786.0 cells was performed using seven miRNA expression vectors (S2 Table) produced by the BLOCK-iT^™^ Pol II miR RNAi expression vector kit (Invitrogen, Waltham, MA, USA) to stably knockdown survivin. Full length survivin expression construct (survivin-tGFP) was obtained from OriGene (OriGene Technologies, Rockville, MD, USA) to constitutively over-express survivin.

Briefly, RCC cells were seeded at a density of 90% confluence for transfection using Lipofectamine^®^ Transfection Reagent (Invitrogen, Waltham, MA, USA) according to manufacturer’s instructions. Antibiotic resistant colonies were analysed using miRNA specific primers (S3 Table) to confirm the presence and correct sequence of the gene of interest. The cells were also lysed for isolation of RNA and protein to analyse the target mRNA and protein expression. The relative expression was obtained by comparing with un-transfected cells and cells stably transfected with the vector alone.

### Cell viability

Generally, 2000–10 000 cells were seeded in clear 96-well flat-bottomed plates and allowed to adhere overnight for 24 h. Cells were then exposed to different concentrations of test compound in growth media for another 72h. *In vitro* cell viability was assessed by MTS assay using the CellTiter 96^®^ AQueous Non-Radioactive Cell Proliferation Assay (Promega Corporation, Fitchburg, WI, USA) and measurements were made on a Benchmark Plus microplate spectrophotometer (Bio-Rad Laboratories, Hercules, CA, USA). Absorbance values from treated cells were expressed as a percentage of control cells to determine the cell growth inhibitory IC_50_. A sigmoidal curve was generated from which IC_50_ (concentration at which 50% of cells are viable) was obtained.

### RNA isolation and quantitative real time polymerase chain reaction

The RNeasy^®^ kit (Qiagen, Hilden, Germany) was used according to manufacturer’s recommendations to isolate high quality total RNA. Complementary DNA (cDNA) was synthesized by reverse transcribing total RNA using the SuperScript^®^ VILO^™^ cDNA Synthesis Kit (Invitrogen, Waltham, MA, USA) according to the manufacturer’s instructions. TaqMan^®^ Fast Advanced Master Mix (Applied Biosystems, Waltham, MA, USA) was used in the detection of PCR products in real time. qRT-PCR was performed using specific DNA probes (S4 Table). Conditions for the Taqman method were 2 minutes at 50°C, 20 seconds at 95°C and then 40 cycles, each consisting of 3 seconds at 95°C and 30 seconds at 60°C. The housekeeping gene used was glyceraldehyde-3-phosphate dehydrogenase (GAPDH). Comparative Ct (cycle threshold) method was used to quantify the expression of the gene of interest.

### Protein analysis using western immunoblotting

Cells were lysed directly in M-PER Mammalian Protein Extraction Reagent (Pierce, Waltham, MA, USA) supplemented with Halt Protease and Phosphatase Inhibitors (Pierce, Waltham, MA, USA). For protein extraction from RCC xenograft tissues, specimens were washed with PBS, minced and homogenized using TissueLyser LT (Qiagen, Hilden, Germany) in T-PER Mammalian Protein Extraction Reagent (Pierce, Waltham, MA, USA) containing Halt Protease and Phosphatase Inhibitors (Pierce, Waltham, MA, USA). Quantitation of proteins was made using the bicinchoninic acid (BCA) method (Pierce, Waltham, MA, USA). Proteins were electrophoresed by SDS-PAGE and transferred onto a nitrocellulose membrane. Blots were incubated with the indicated primary antibodies (Cell Signaling Technology Inc, Danvers, MA, USA) and horse-radish peroxidase conjugated secondary antibodies. All proteins were visualised with Immobilon chemiluminescent detection reagent (Millipore, Billerica, MA, USA).

### Transcriptome analysis

RCC786.0 cells were treated with control (0.1% DMSO) or YM155 (40nM or 80nM) for 24 h and 48 h. RNA was isolated, quantified using BioSPEC-Mini (Shimadzu) and its quality evaluated using the Agilent 2100 Bioanalyzer (Agilent Technologies). The samples were processed using Affymetrix 3’ IVT Express to create biotin-labeled amplified RNA, which was subsequently fragmented and hybridized to Affymetrix PrimeView Human Gene Expression Arrays (16 hours, 45°C, 60 rpm). Arrays were scanned using an Affymetrix 3000 7G scanner. Data processing was conducted on the Partek Genomics Suite V6.3 (Partek, St Louis, MI). The raw expression values obtained were processed using the Robust Multi-chip Average (RMA) method [33]. p values for individual genes were adjusted using the Benjamini and Hochberg method. A list of significantly perturbed genes (p<0.05 and fold change >2) was generated and pathway analysis conducted using the Ingenuity Pathway Analysis (IPA) knowledge base. The significance of the association between the input data set and the functions or pathways was determined by two parameters: (i) the ratio of the number of genes from the data set that map to the function/pathway divided by the total number of genes that map to the function/pathway and (ii) p-value calculated using Fisher’s exact test to determine the probability that the association between the genes in the dataset and the function/pathway arose by chance alone.

### Statistical analysis

GraphPad Prism version 5.0 (GraphPad Software Inc., San Diego, CA, USA) and Excel (Microsoft Corporation, Redmond, WA, USA) were used to plot and analyse data. Graphs were plotted to show mean values and error bars which depicted standard error of the mean (SEM). The student’s t-test and analysis of variance (ANOVA) with Bonferroni post–hoc test were used for the comparison of mean values between two and multiple (>2) groups, respectively. A minimum of 95% level of significance (p<0.05) was used to indicate statistical significance. All experiments were repeated at least twice.

## Results

### RCC cell lines without functional VHL protein express higher levels of survivin but silencing survivin with siRNAs arrest colony formation in both VHL-positive and VHL-null RCC cell types

**Fig 1:**
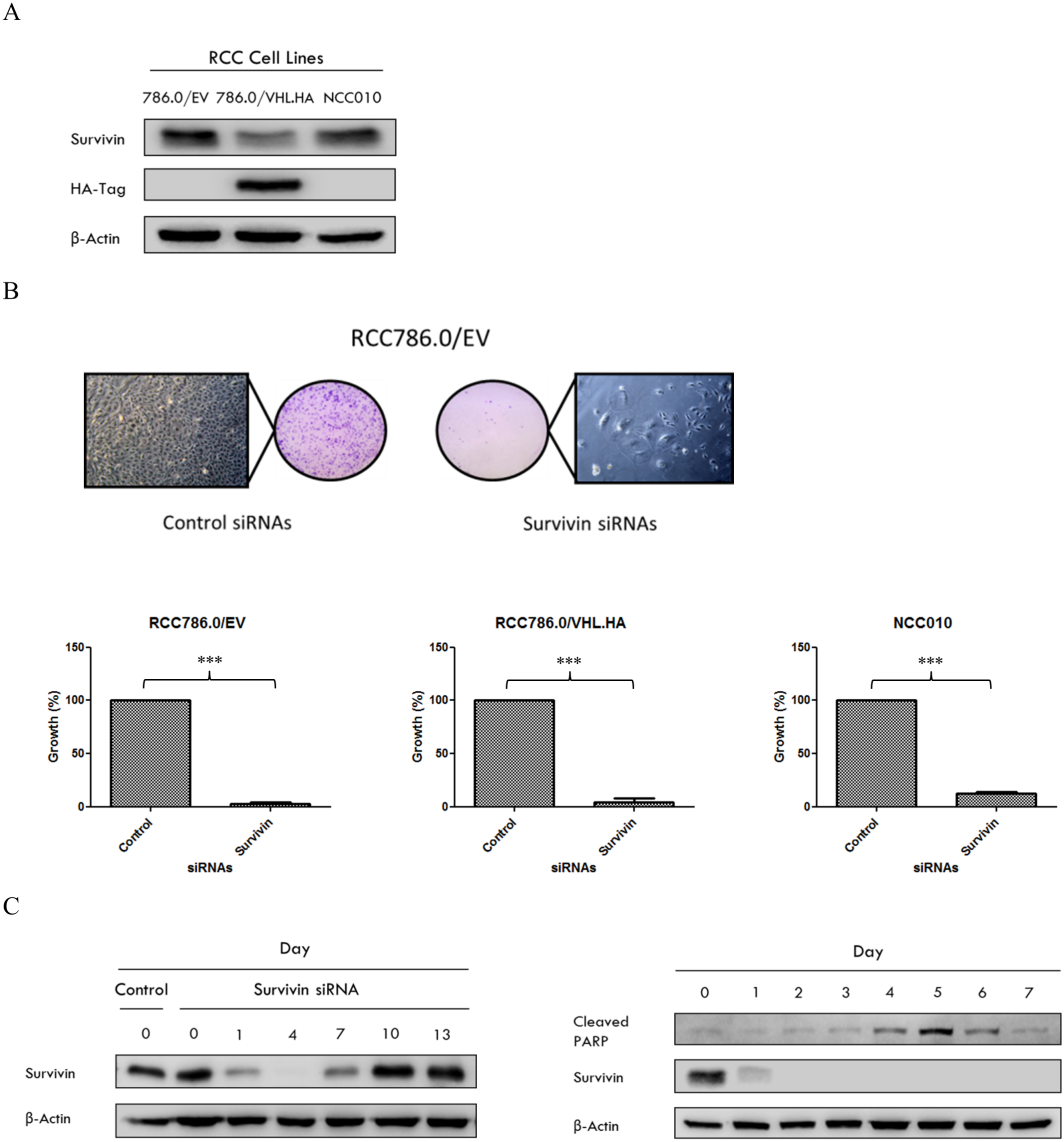
(A) Immunoblots of whole cell lysates of RCC786.0/EV, RCC786.0/VHL.HA and NCC010. β-actin is the loading control. HA-tag indicates the presence of the VHL transgene. (B) The effect of gene silencing on the proliferation of these cells was assessed by clonogenic survival assay. Visible and discrete colonies of transfected RCC786.0/EV were counted at the end of 10–14 days (100X magnification). The growth of RCC786.0/EV, RCC786.0/VHL.HA and NCC010 cells post-transfection are depicted and expressed as a % of the control. *** p<0.001 from corresponding control. (C) The expression of survivin in RCC cells transfected with survivin siRNAs was monitored from Day 0 to Day 13. The degradation of survivin siRNAs started after day 4 and the re-expression of survivin was observed from day 7 within the cellular environment. The expression of cleaved PARP, a marker of apoptosis in RCC786.0/EV cells following survivin siRNA-induced gene silencing was time dependent. Cleaved PARP appears on Day 4, approximately 48 h after expression of the survivin protein was inhibited on Day 2. There is a difference in the expression of survivin at Day 7 of the siRNA transfected cells in Fig 1C (left and right hand panels). This is attributed to the slight difference in transfection efficiencies across independent batches of transfected cells.

We first determined the levels of survivin in a panel of human clear cell RCC cell lines comprising a patient-derived cell line NCC010 and an isogenic pair (RCC786.0/EV, RCC786.0/VHL.HA). Immunoblotting with anti-HA-tagged antibody confirmed the VHL status of RCC786.0/EV which is VHL-null whereas its isogenic derivative RCC786.0/ VHL.HA is VHL positive (Fig 1A). The primary NCC010 cell line was found to be VHL-null. Although survivin was detected in all three cell lines, we noted significantly higher levels of survivin in the VHL-null cells (RCC786.0/EV, NCC010) as compared to VHL-positive RCC786.0/VHL.HA cells. These results hint at a possible link between VHL status and survivin expression levels, with VHL-null cells associated with higher survivin content.

Before investigating the cytotoxic effects on YM155 on the afore-mentioned RCC cell lines, we sought to confirm their dependence on survivin for viability. To this end, survivin specific siRNAs were introduced to RCC786.0/EV, RCC786.0/VHL.HA and NCC010 cells to suppress survivin expression (S1 Fig). The survivability of the transfected cell lines were then assessed in the clonogenic cell survival assay [34] (Fig 1B). The results showed a staggering 95% loss in colony forming capacities in all the transfected cell lines regardless of VHL status.

Next, we asked if apoptosis was involved in the dramatic loss in viability of the survivin silenced cells. To address this question, we first assessed the stability of the siRNA-silenced survivin gene in transfected RCC786.0/EV cells over 13 days (Fig 1C). The results showed that a single transfection achieved >90% (quantified by densitometry) knockdown of survivin by day 4 which lasted up to day 7. Thereafter, a rebound in survivin levels was observed, likely due to the degradation of the siRNAs and re-expression of survivin in the cellular environment. Having established the time interval within which survivin was suppressed in the transfected cells, we monitored the expression of cleaved PARP (an apoptotic marker protein) [35] over the same period. Fig 1C shows detectable levels of cleaved PARP levels at days 4 to 6, with a peak on day 5. Thereafter, there was a visible decline in cleaved PARP levels. Notably, increases in cleaved PARP coincided with low levels of survivin in the survivin-silenced cells and vice versa, a trend which vindicated the involvement of apoptosis. Thus, we have confirmed that silencing survivin adversely affected survivability of VHL positive and VHL null RCC cell lines and that apoptosis was involved in the demise of the survivin-silenced RCC786.0/EV cells.

### The survivin suppressant YM155 potently reduces viability of patient derived and immortalized RCC cell lines regardless of VHL status

Having shown that survivin was a critical determinant of RCC cell viability, we proceeded to investigate the growth inhibitory activity of YM155 on an extended panel of RCC cell lines. These were the previously investigated cell lines (NCC010, RCC786.0/EV, RCC786.0/VHL.HA), two additional patient derived cells (NCC035, P.RCC) and three immortalised cell lines (RCC786.0, ACHN, A498). These cells are of clear cell histological subtype except for P.RCC, which is a papillary RCC. The VHL status of NCC010, RCC786.0/EV, RCC786.0/VHL.HA was mentioned earlier (Fig 1A, VHL-null except for RCC786.0/VHL.HA). The other patient derived cell line (NCC035) was also VHL-null (personal communication from Prof Teh Bin Tean, National Cancer Centre, Singapore) while the VHL status of the remaining cell lines (commercially purchased) were based on the supplier’s reports. Of these, RCC786.0 and A498 lacked a functional pVHL whereas ACHN and P.RCC were VHL positive.

**Fig 2:**
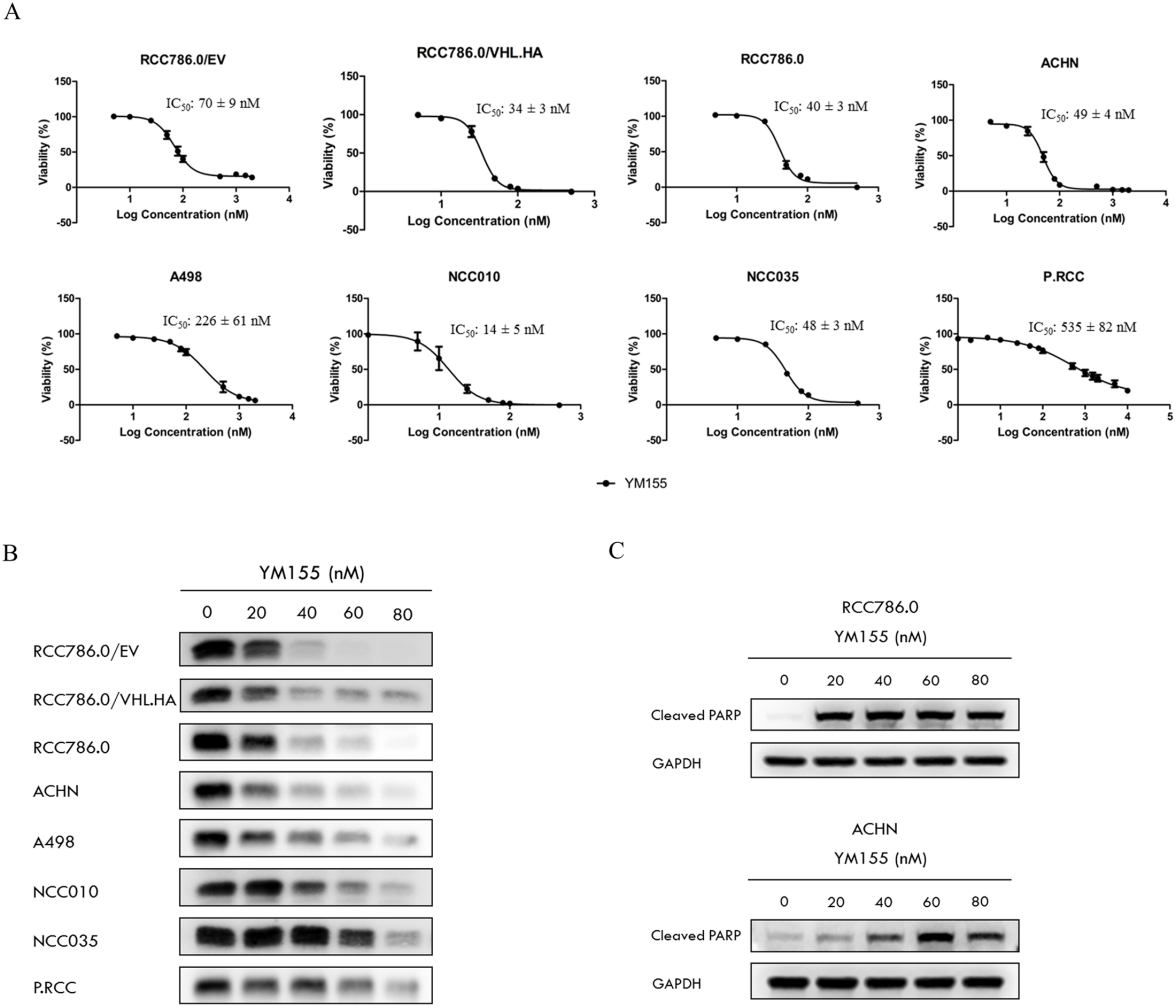
The effects of YM155 on RCC cells. (A) Dose-response curves of YM155 on a panel of RCC cell lines with varying VHL status. Cells were incubated with YM155 for 72h. RCC786.0/EV, RCC786.0, NCC010, NCC035 and A498 are VHL null cell lines. RCC786.0/VHL.HA, ACHN and papillary RCC (P.RCC) are VHL positive cell lines. NCC010, NCC035 and P.RCC are patient derived RCC cell lines while the remaining RCC cell lines are commercially available. YM155 exhibited nanomolar growth inhibitory activity on these RCC cells but was significantly less potent on P.RCC. (B) Survivin levels in various RCC cell lines treated with increasing concentrations (20 nM– 80 nM) of YM155 for 48 h. YM155 effectively down-regulated survivin in a dose dependent manner, albeit to varying degrees depending on the cell type. (C) RCC786.0 and ACHN were treated with increasing concentrations (20 nM– 80 nM) of YM155 for 72h. YM155 effectively up-regulated cleaved PARP in a dose dependent manner.

Fig 2A shows that YM155 adversely affected viabilities of all the investigated cell lines at nanomolar concentrations. There were indications that the growth inhibitory potency of YM155 was greater on VHL positive cells, as seen from the isogenic pair of RCC786.0 cells where the VHL null 786.0/EV was less susceptible to YM155 (IC_50_ 70 nM) compared to its VHL positive derivative 786.0/HA.VHL (IC_50_ 34 nM). Similarly, YM155 was less potent (IC_50_ 226 nM) on the VHL-null A498 cells and more potent on the VHL positive ACHN cells (IC_50_ 49 nM). On the other hand, we found strikingly comparable YM155 potencies in the parental RCC786.0 (IC_50_ 40nM) and transformed RCC786/HA.VHL (IC_50_ 34nM), despite their different VHL status. Interestingly, the papillary RCC subtype P.RCC is VHL positive but malignancy is not VHL dependent [36,37] was the least susceptible to YM155 (IC_50_ 535nM). Taken together, YM155 preferentially targets clear cell malignancies of the kidney as compared to other histological subtypes.

We also probed the dose dependent effects of YM155 on survivin levels in the treated cells (Fig 2B). As anticipated, YM155 reduced the expression of survivin across the entire panel of RCC cells. However, as in cell viability, there was no firm indication that the extent of survivin suppression was influenced by the presence or absence of VHL. Thus, we noted that YM155 at 60nM completely abolished survivin expression in the VHL-null, high survivin RCC786.0/EV cells (Fig 1A), but only partially on NCC010 which is also VHL null and high survivin-expressing.

YM155 also induced apoptosis in RCC786.0 (VHL null) and ACHN (VHL positive) cell lines. As seen from Fig 2C, YM155 caused a dose dependent increase in the apoptotic marker protein cleaved PARP in both cell lines. Thus, the potent effects of YM155 on the viability of RCC cell lines were mediated by apoptosis, occurred independently of the VHL status and the level of survivin in the affected cells.

### Growth inhibitory activity of YM155 is unaltered in RCC cells in which survivin expression has been stably silenced or overexpressed

To further interrogate the role of survivin in the growth inhibitory activity of YM155, we prepared clones of RCC786.0 in which the survivin gene was stably silenced or overexpressed. The premise was that if YM155 affected the viability of these clones to the same extent as parental RCC786.0 cells which have constitutive survivin levels, it would indicate that growth inhibition by YM155 is not exclusively mediated by survivin.

**Fig 3:**
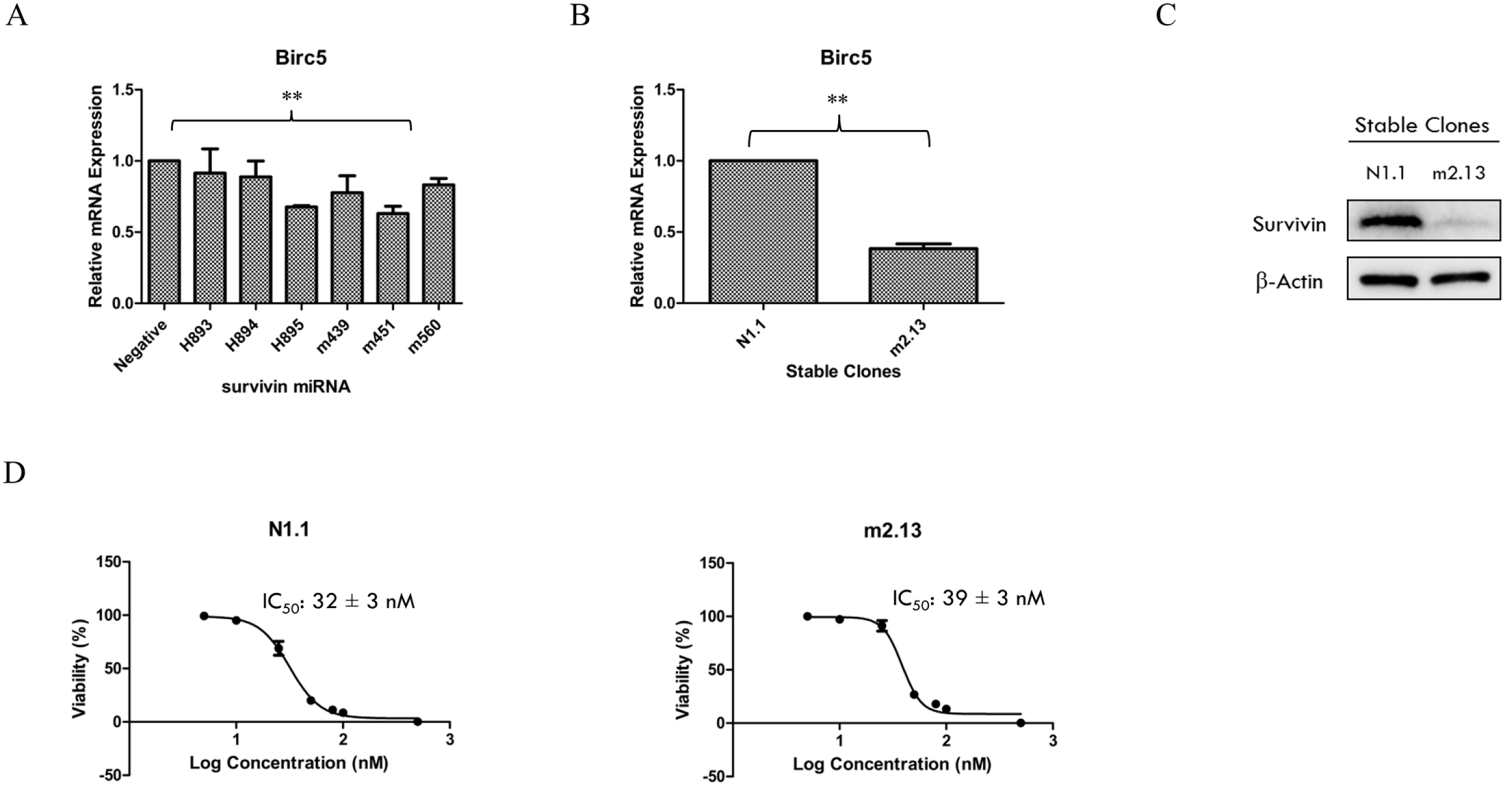
Survivin knockdown stable clones. (A) m451 plasmid achieved the greatest effect (37% knockdown) on the silencing of the survivin mRNA expression after transient transfection (transfection efficiency ~80%) when compared to the negative control plasmid. ** p < 0.01 from corresponding control. (B) m2.13 clone constitutively expressed survivin miRNA which cleaved survivin mRNA transcripts produced inside the cell and led to a survivin mRNA expression of only 38% compared to the negative clone, N1.1. ** p < 0.01 from corresponding control. (C) The protein expression of survivin in m2.13 was significantly lower compared to N1.1. (D) The growth inhibitory IC_50_s of YM155 on these clones were similar.

A clone of RCC786.0 in which the translational capacity of the survivin gene has been suppressed was prepared. Fig 3A shows that the m451 plasmid “silenced” mRNA expression to the greatest extent (37% knockdown, p<0.001) after transient transfection (transfection efficiency ~80%) as compared to the negative control plasmid. A stable clone of RCC786.0 which constitutively expressed the survivin miRNA and a control clone which expressed the negative control plasmid were generated. m2.13 was selected as the most satisfactory survivin knockdown clone. Fig 3B shows that the m2.13 clone constitutively expressed survivin miRNAs that cleaved cellular survivin mRNA transcripts and reduced survivin mRNA expression to only 38% of that found in the negative control clone N1.1. The protein expression of survivin in m2.13 was also significantly lower compared to N1.1 (Fig 3C). The growth inhibitory IC_50_ of YM155 on m2.13 was determined (39 nM) and found to be comparable to that of N1.1. (IC_50_ 32nM). Clearly, differences in survivin mRNA and protein expression levels did not affect the vulnerability of cells to YM155 (Fig 3D).

**Fig 4:**
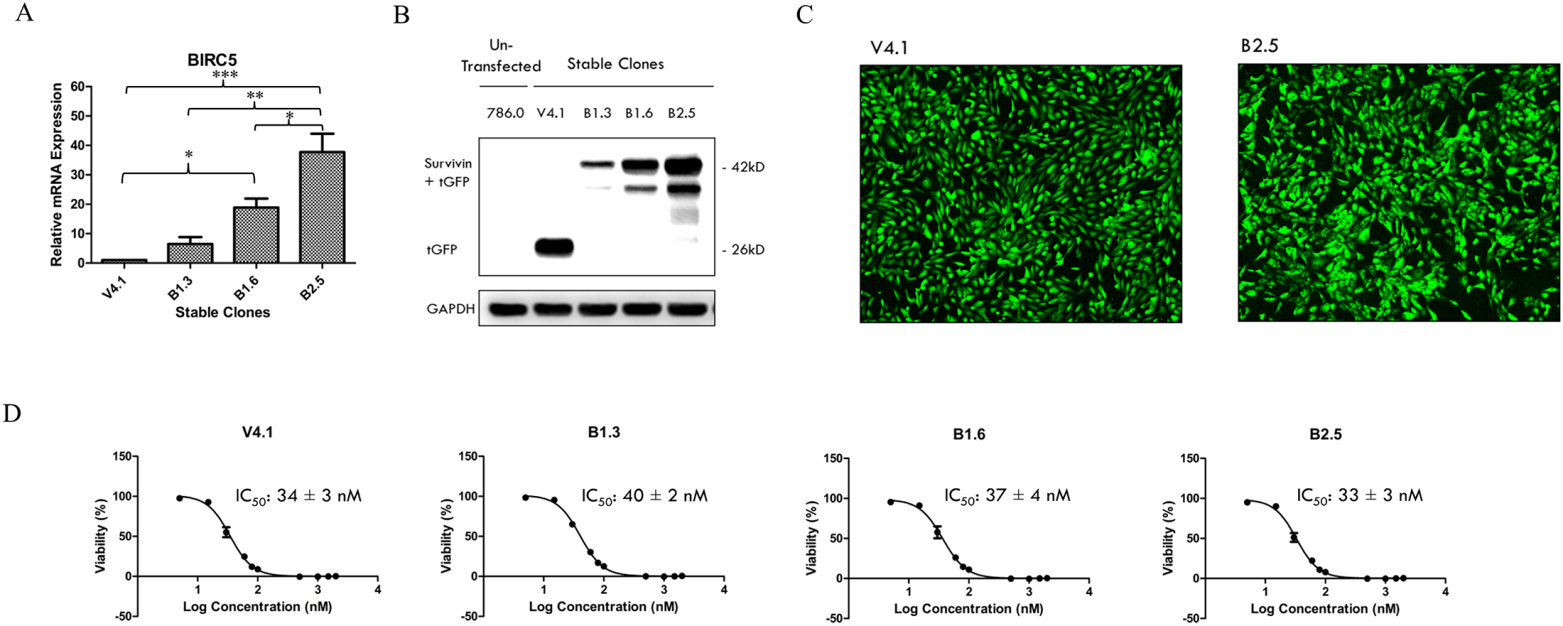
Survivin over-expressed stable clones. (A) B1.3, B1.6 and B2.5 constitutively over-expressed BIRC5 mRNA when compared to the control clone V4.1 which contains the expression vector without the BIRC5 gene insert. Among these clones, B2.5 increased amplification of the BIRC5 mRNA to the greatest extent (~38 fold). * p <0.05, ** p < 0.01 and *** p < 0.001 from corresponding control. (B) B1.3, B1.6 and B2.5 constitutively over-expressed survivin protein when compared to the control clone V4.1. The control clone V4.1 which contains the expression vector without the BIRC5 gene insert only expressed tGFP (26kD). Among the clones, B2.5 increased protein expression of survivin tagged with tGFP (42kD) to the greatest extent. (C) Visualisation of the C-terminal tGFP tag under a fluorescence microscope (100X) enables positive selection of transfected cells for stable cell line production. (D) The growth inhibitory IC_50_s of YM155 on these clones were similar.

Next, we proceeded to investigate the effects of survivin overexpression on the growth inhibitory properties of YM155. RCC786.0 cells were stably transfected with a ready-to-use expression vector which contained a *BIRC5* gene insert tagged with a C-terminal tGFP to generate a stable expression clone that constitutively expressed the survivin mRNA and protein. An expression clone which contains the expression vector without the *BIRC5* gene insert was used as control (V4.1). *BIRC5* gene expression was assessed in the various clones (B1.3, B1.6, B2.5) (Fig 4A) and found to be highest in B2.5. *BIRC5* mRNA levels were 38 fold higher in this clone when compared to the control V4.1 clone (p < 0.001). It also has a higher survivin expression than the control clone (Fig 4B). In spite of higher levels of survivin mRNA and protein expression, the growth inhibitory potency of YM155 on B2.5 was comparable to the control clone which does not over-express survivin. Thus, there is corroboratory evidence from both knockdown and overexpression experiments that the growth inhibitory activity of YM155 in RCC cell lines is not exclusively mediated by its suppression of survivin.

### Transcriptome analysis identifies tumour suppressors *CYLD*, *FOXO1* and tumour promoter *ID1* as targets of YM155 in RCC786.0 cells

**Fig 5:**
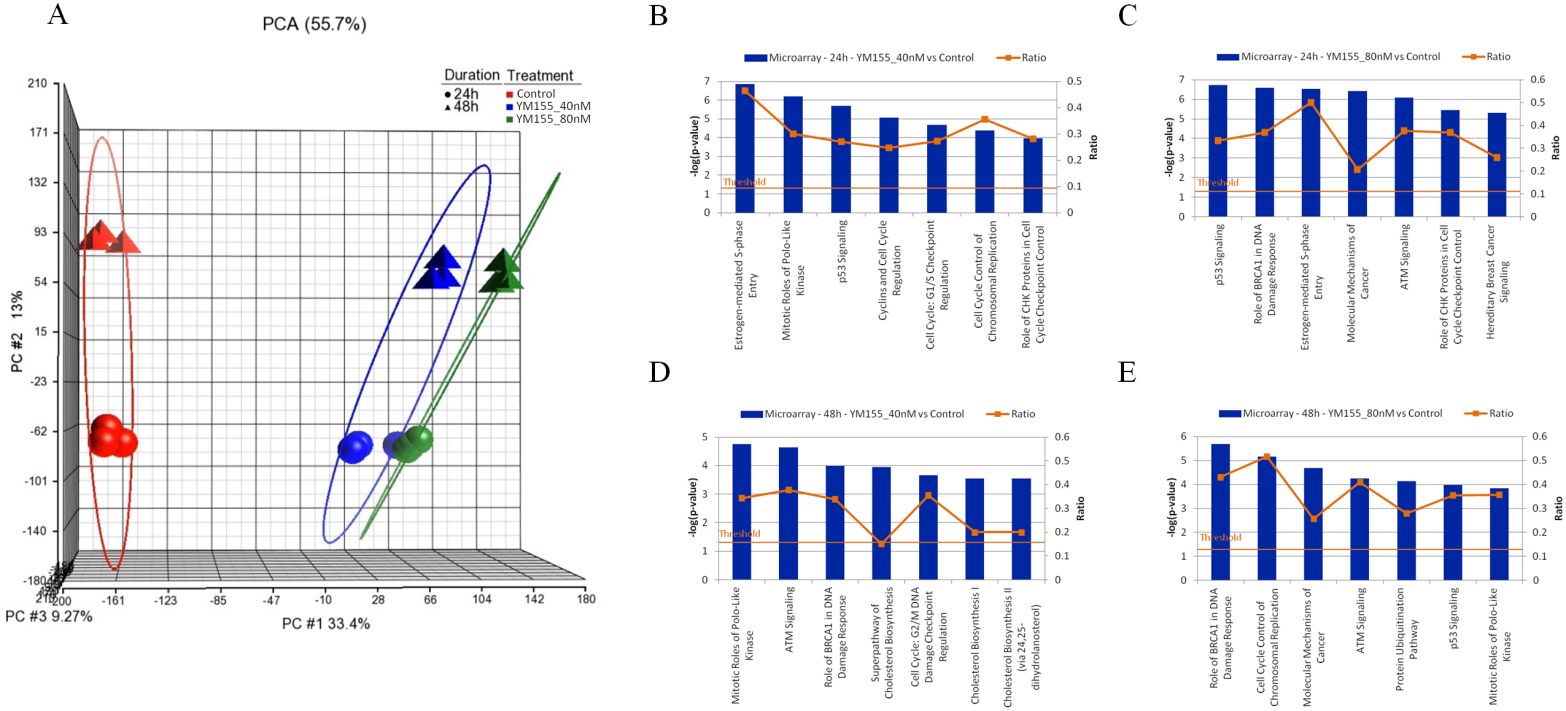
Microarray analysis on RCC786.0 cells treated with 40nM or 80nM of YM155 for 24 h or 48 h. (A) PCA clustering analysis of the results showed significant differences between treated and control cells. No significant difference was observed between the triplicate runs for each treatment arm indicating good reproducibility. Cells treated for 24 h and 48 h showed different gene expression profiles. Cells exposed to different concentrations of YM155 did not show significant differences in gene expression at either time point. Analyses were carried out on Partek (Partek Inc., Missouri, USA). Graphical representation of the top canonical signaling pathways in YM155-treated RCC786.0 cells that were significantly affected in the presence of (B) YM155, 40 nM, 24 h; (C) YM155, 80 nM, 24 h; (D) YM155, 40 nM, 48 h; and (E) YM155, 80 nM, 48 h.

Results from the preceding sections point to the involvement of other molecular targets in the growth inhibitory activity of YM155. To have a better understanding of the scope and range of targets involved, we carried out a transcriptome analysis of RCC786.0 cells that were treated with YM155 at two concentrations (40 nM and 80 nM) for 24 h or 48 h. The resulting gene expression profiles were analysed by principal component analysis (PCA) (Fig 5A). We detected a distinct clustering of gene expression profiles based on the concentrations of YM155 but not on treatment times, indicating that changes in gene expression were preferentially affected by the length of exposure to YM155 rather than the amount exposed. The gene expression profiles at each treatment condition were then analyzed to identify members that were significantly altered by 2 fold or more (adjusted p value < 0.05). Pathway analyses of these shortlisted genes were undertaken to identify the relevant canonical signaling pathways in which their protein products would be found. We noted that p53 signaling, ATM signaling and cell cycle regulation which are components of the p53 connection pathway, were highly ranked in all the treatment arms (Fig 5B–5E). The protein products in this pathway are involved in activating repair mechanisms when DNA is damage, arrest growth by holding the cell cycle at the G_1_/S regulation point to enable repair, and initiate apoptosis if DNA damage is irreparable. We then selected representative genes from the short list of 108 genes that have been reported to play a role in the pathogenesis of RCC. They are *CYLD*, *FOXO1*, *ID1 and BIRC5*. *CYLD* is a deubiquitinating enzyme that negatively regulates NF-κB transcription factor activity [38]. Activated NF-κB can upregulate the transcription of various IAPs including survivin to suppress apoptosis [39]. Human HCC cell line infected with CYLD expression plasmid can reverse the increase in survivin expression [40]. *FOXO1* is a transcription factor that acts as a tumour suppressor, mediates cell cycle arrest and promotes apoptosis [41,42]. *FOXO1* mediates the transcription of survivin and siRNA knockdown of *FOXO1* had been reported to enhance survivin expression, thereby inhibiting apoptosis [43,44]. *ID1* is an inhibitor of DNA binding that interacts and inhibits the transcriptional activation ability of basic HLH proteins which leads to tumour growth and angiogenesis [45]. Knockdown of ID1 has been shown to significantly decrease mRNA and protein levels of survivin in colorectal cancer cells [46] as well as to remove survivin inhibition of apoptosis in head and neck squamous cell carcinoma [47]. *BIRC5* encodes the expression of anti-apoptotic protein survivin and elevated activity of this survival cascade has been implicated in cancer cell survival and disease progression [48,49,50]. The gene profiles indicated that YM155 down-regulated *ID1* and *BIRC5* which are tumour promoters, and upregulated *CYLD* and *FOXO1* which are tumour suppressors.

**Fig 6:**
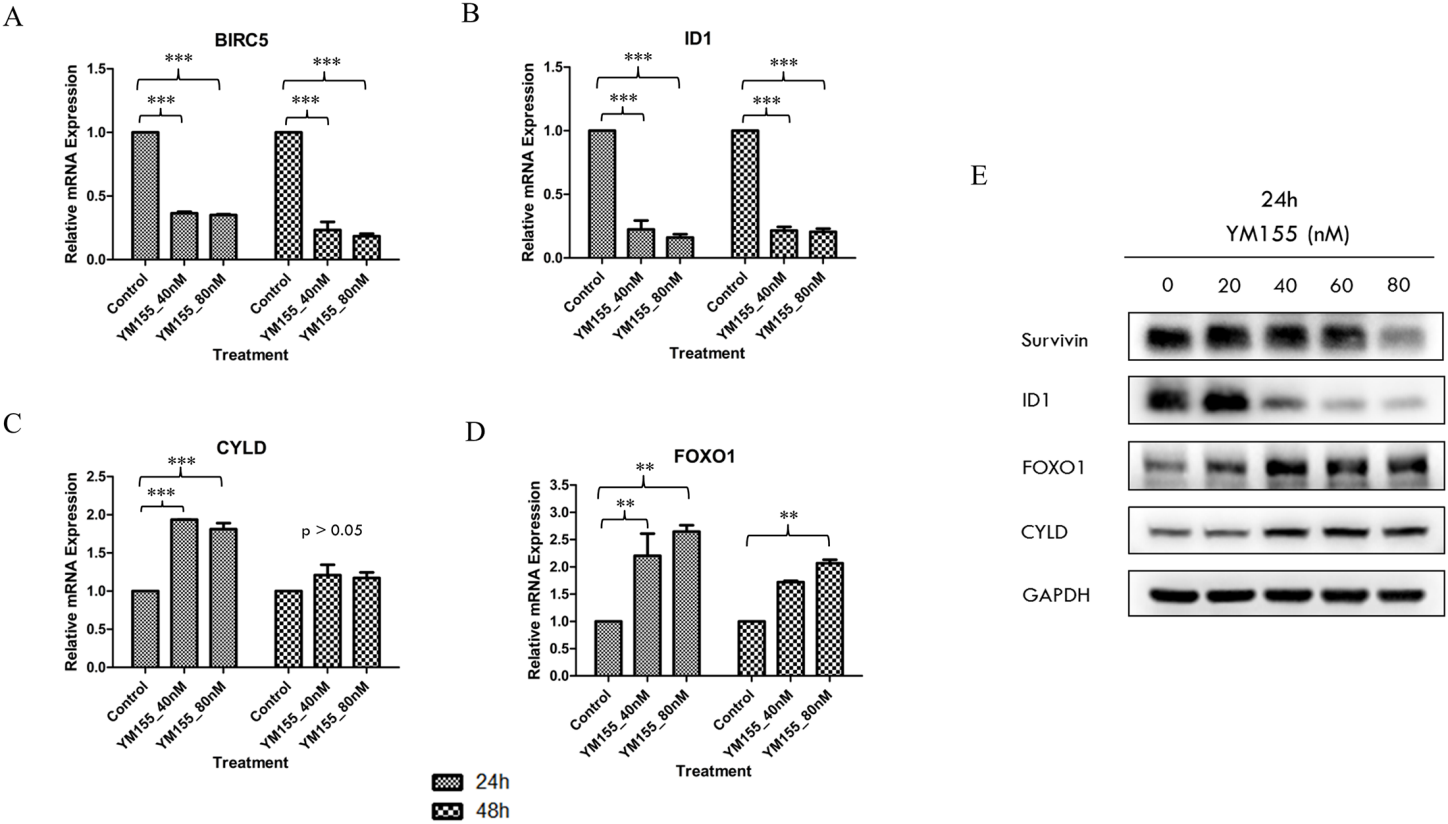
Validation of selected genes associated with p53 connection pathway was carried out using quantitative real-time polymerase chain reaction (qRT-PCR) and western immunoblotting. (A) BIRC5 mRNA levels were reduced by ~2.8 fold at 24 h and ~5 fold at 48 h at both test concentrations. (B) ID1 mRNA levels were reduced by ~5 fold at both test concentrations and time points. (C) CYLD mRNA levels were increased by ~2 fold at 24 h but returned to basal levels at 48 h at both test concentrations. (D) FOXO1 mRNA levels were increased by ~2.4 fold at 24 h and ~1.8 fold at 48 h at both test concentrations. ** p<0.01, *** p<0.001. (E) The protein expression of survivin reduced slightly while a dose-dependent decrease in ID1 and increase in FOXO1 and CYLD were observed with 24 h exposure to YM155 in RCC786.0 cells.

We then proceeded to determine the mRNA levels of *FOXO1*, *ID1*, *BIRC5* and *CYLD* by quantitative real-time polymerase chain reaction (qRT-PCR) in RCC786.0 cells that were treated with YM155 under conditions similar to those employed in the transcriptome analysis (Fig 6A–6D). In keeping with the preceding results, we found that mRNA expression levels of *ID1* and *BIRC5* were elevated in treated cells versus control, whereas those of *CYLD* and *FOXO1* were decreased relative to control. On the other hand, changes in mRNA expression levels did not differ significantly in cells treated with 40 nM or 80 nM YM155, which was consistent with the PCA clustering pattern. The effect of treatment time on mRNA expression varied among the genes. In the case of *ID1*, the effect of time was minimal and mRNA levels were reduced to the same extent (5 fold) at both time points (24 h, 48 h). In contrast, *BIRC5* mRNA levels showed a distinct time dependency and there was a greater loss in mRNA levels (5 fold) at 48 h than at 24 h (2.8 fold). As for genes (*CYLD*, *FOXO1*) that were upregulated, mRNA levels were elevated only at the 24 h time point (2 fold for *CYLD*, 2.4 fold for *FOXO1* compared to controls). After 48 h, *CYLD* mRNA levels returned to basal levels whereas *FOXO1* mRNA levels remained elevated but declined with time (1.8 fold at 48 h compared to 2.4 fold at 24 h).

To validate the changes in mRNA expression levels of the four genes, we treated RCC786.0 cells with a range of YM155 concentrations (20 nM–80 nM) for 24 h and monitored their protein products by protein immunoblotting. The results, as shown in Fig 6E, were confirmatory. YM155 suppressed the expression of both survivin and ID1, with a noticeably more pronounced effect on ID1 which was reduced at 40 nM YM155, in contrast to survivin which required 80 nM YM155 before a decrease was observed. However, FOXO1 and CYLD were equally susceptible to YM155 and increases in their protein levels were observed at 40 nM YM155.

### The combination of YM155 and sorafenib displays synergistic growth inhibitory activity on RCC786.0 cells but failed to suppress growth of a patient-derived RCC xenograft model

**Fig 7:**
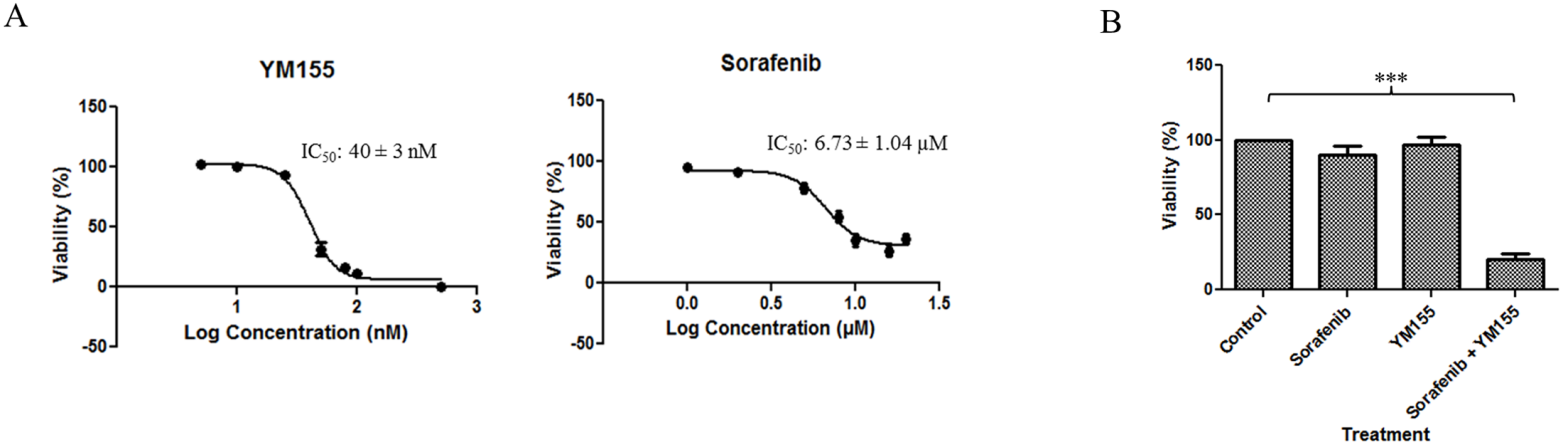
Growth inhibitory activity of YM155 and sorafenib alone and in combination. In MTS cell proliferative assay, (A) the IC_50_ of YM155 was 40 ± 3 nM. Sorafenib had an IC_50_ of 6.73 ± 1.04 μM but the growth inhibitory effect does not go below 64%. (B) Drugs in combination at ½ x IC_50_ reduced cell viability to ~20% as compared to the negligible effects elicited by either drug used alone at the same concentration. *** p < 0.001 compared to control.

Several clinical studies have shown that monotherapy with YM155 has limited efficacy [26,51] and have recommended combination therapy with other anti-cancer drugs as the preferred option [28,52,53,54,55]. Earlier, YM155 was reported to augment the activity of temsirolimus, a widely employed targeted therapy for RCC, in xenograft models [56]. The tyrosine kinase inhibitor sorafenib is widely endorsed as a standard of care for RCC and an alternative to temsirolimus. Dose reduction is frequently required in patients treated with sorafenib due to its side effects. Combining YM155 with a lower dose of sorafenib may be a means of averting this undesirable outcome, if the combination of both drugs can be shown to be synergistic. To this end, we first determined the growth inhibitory IC_50_ concentrations of YM155 and sorafenib on RCC786.0 cells (Fig 7A). The growth inhibitory effects of four combinations of the two drugs were explored, namely (i) both drugs at their IC_50_ concentrations; (ii) both drugs at ½ x IC_50_ concentrations; (iii) YM155 at its IC_50_ and sorafenib at ½ x IC_50_; and (iv) YM155 at ½ x IC_50_ and sorafenib at its IC_50_. The combination index (CI) of each treatment was then determined by the Chou and Talalay equation [57] to identify combinations that were synergistic (CI < 0.9).

As seen from Table 1, synergy was observed when YM155 and sorafenib were combined at concentrations equivalent to ½ x IC_50_. This combination reduced cell viability to ≈ 20% as compared to the negligible effects elicited by either drug used alone at the same concentration (Fig 7B).

**Table 1:** Combination indices (CI) of YM155-sorafenib combinations.

	½ IC_50_ YM155 ½ IC_50_ sorafenib	IC_50_ YM155 IC_50_ sorafenib	IC_50_ YM155 ½ IC_50_ sorafenib	½ IC_50_ YM155 IC_50_ sorafenib
**CI** ^a^	0.80 ± 0.07	1.51 ± 0.05	1.06 ± 0.04	1.24 ± 0.11

^a^ Determined using the Chou and Talalay Equation. CI < 0.9 is synergistic; CI of 0.9 to 1.1 is additive and CI > 1.1 is antagonistic.

**Fig 8:**
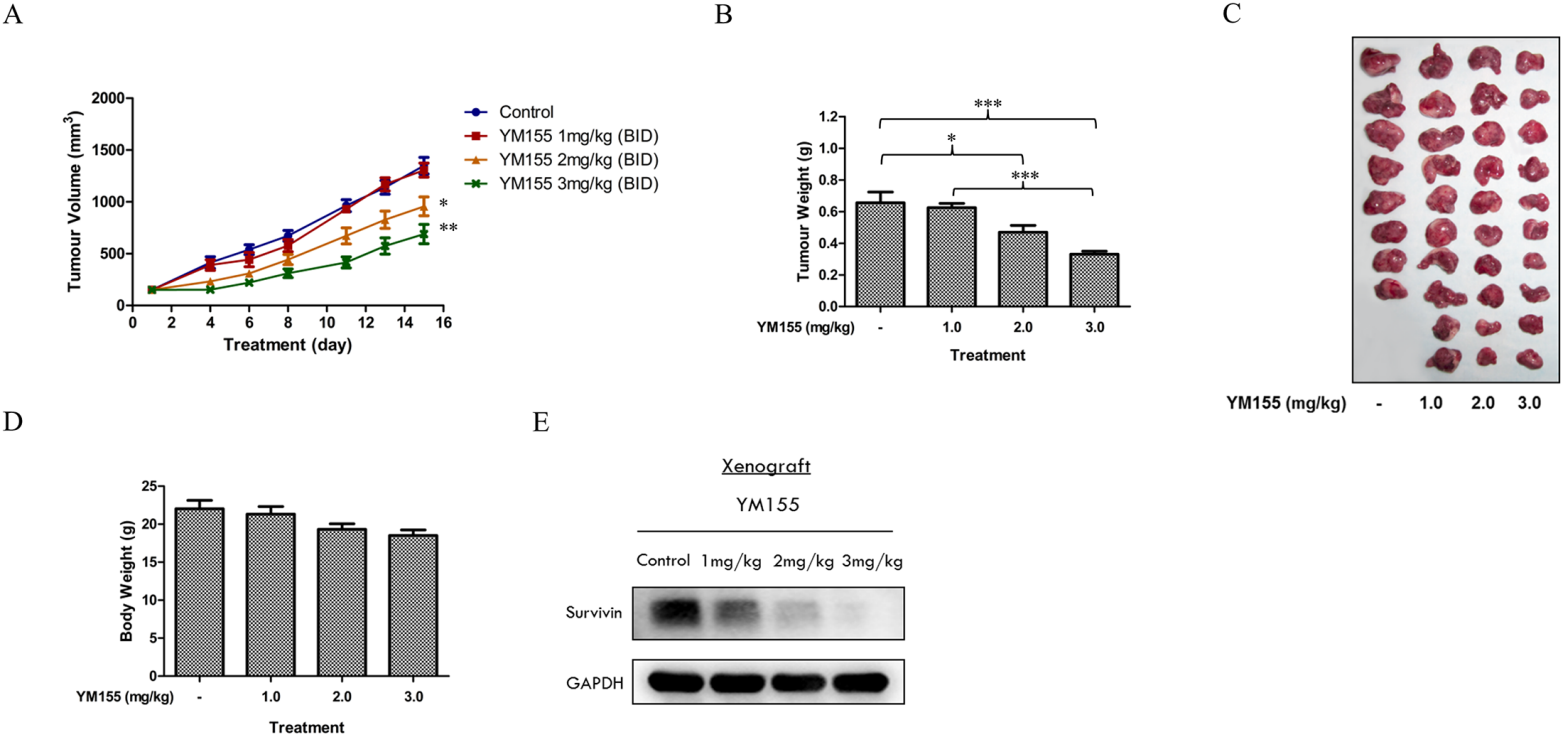
In vivo assessment of YM155 using a patient-derived RCC xenograft model in SCID mice. Mice bearing RCC-19-0809 tumours were randomised (10 mice per group) and treated with vehicle (PBS) or increasing doses of YM155 (1 mg, 2 mg and 3 mg/kg twice a day) for 15 days. (A) The mean tumour volume of vehicle- and YM155- treated mice at the given time-points showed a decrease in the rate of tumour growth at higher doses of YM155. (B) The mean tumour weight of vehicle- and YM155- treated mice on the day of animal sacrificed. (C) A picture representative of vehicle- and YM155 treated tumours. (D) The mean body weight of the mice in response to the treatment showed that YM155 was well tolerated in tumour bearing animals with no significant decrease in body weight at the end of the treatment period. (E) Lysates of 3 tumours from each group were pooled and subjected to western immunoblotting analysis. Representative blots shown indicated a dose-dependent reduction in the protein expression of survivin in the tumours of YM155-treated mice. * p < 0.05 and *** p < 0.001 using one-way anova/Bonferroni post-hoc test.

Having shown that a combination of YM155 and sorafenib was potentially synergistic, we proceeded to evaluate the YM155-sorafenib combination on a murine xenograft that was derived from a nephrectomised patient with clear cell RCC. To determine a suitable dose of YM155 for the combination, we first treated the xenograft-bearing SCID mice with YM155 at 1, 2 or 3 mg/kg, IP, twice daily for 15 days. An earlier study had shown that these doses were well-tolerated in normal SCID mice (results not shown). When the tumour bearing mice were dosed at these levels, we found that YM155 caused significant reductions in tumour volumes and tumour weights at 2 mg/kg and 3 mg/kg (Fig 8A–8C) without eliciting pronounced weight loss in the treated animals (Fig 8D). Immunoblotting of lysates prepared from excised tumours showed that the slowing of tumour growth was accompanied by reductions in survivin levels.

**Fig 9:**
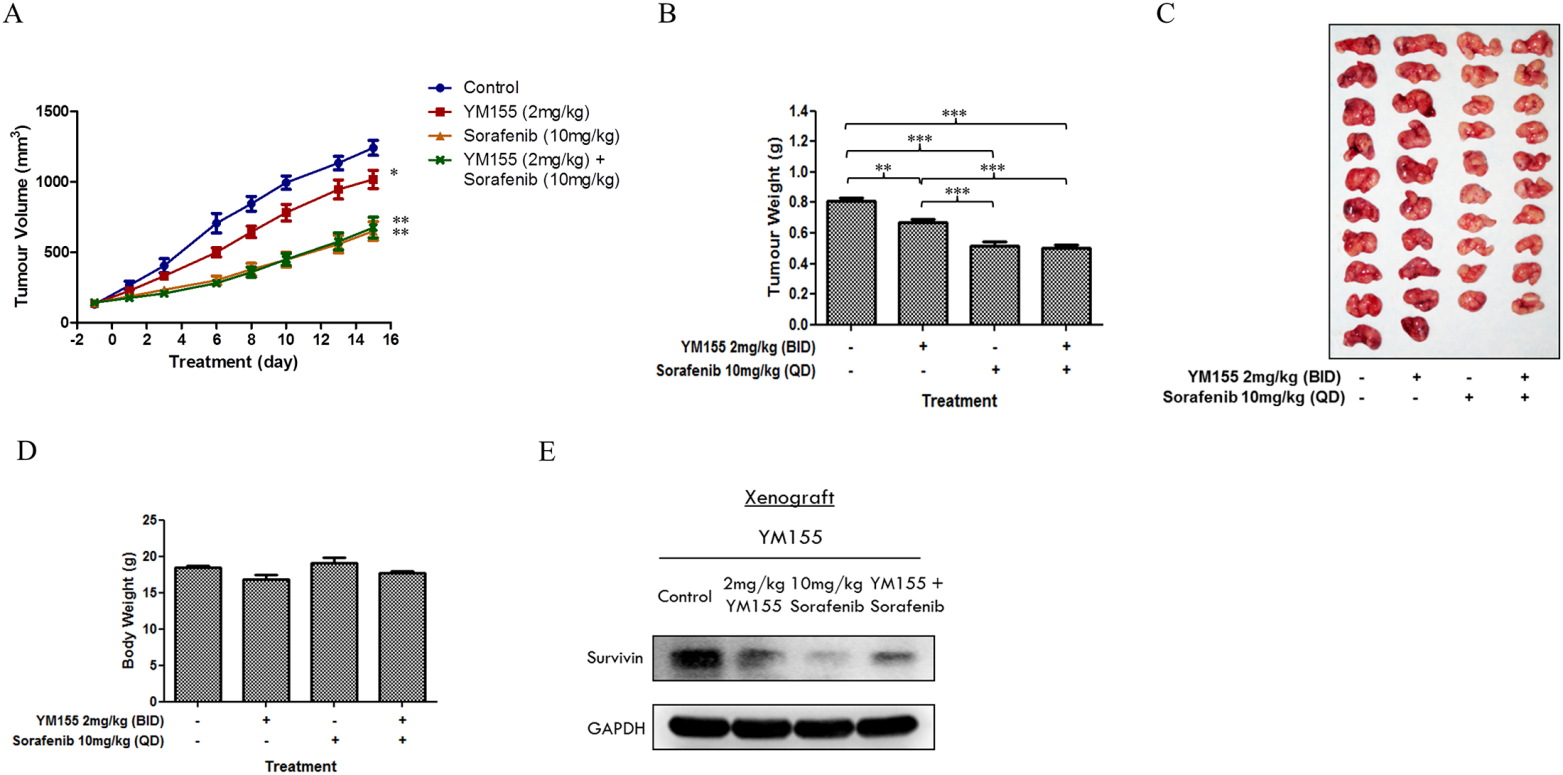
In vivo assessment of combination of YM155 and sorafenib using a patient-derived RCC xenograft model in SCID mice. Mice bearing RCC-19-0809 tumours were randomised (10 mice per group) and treated over a period of 15 days with either (i) vehicle, (ii) YM155 (2 mg/kg twice a day), (iii) sorafenib (10 mg/kg once a day), or (iv) YM155 (2 mg/kg twice a day) and sorafenib (10 mg/kg once a day). (A) The mean tumour volume of vehicle-, YM155-, sorafenib- and (YM155-sorafenib)- treated mice at the given time-points showed a decrease in the rate of tumour growth when compared to the vehicle control. However, there was no significant difference between treatment with sorafenib alone and in combination with YM155. (B) The mean tumour weight of control and treated mice on the day of animal sacrificed. There was a significant difference between YM155 alone and sorafenib alone, but no significant difference between sorafenib alone and in combination with YM155. (C) A picture representative of control and treated tumours. (D) The mean body weight of the mice in response to the treatment showed that all treatments were well tolerated with no significant decrease in body weight at the end of the treatment period. (E) Lysates of 3 tumours from each group were pooled and subjected to western immunoblotting analysis. Representative blots shown indicated a reduction in the protein expression of survivin in the tumours of all treated mice. However, combined YM155-sorafenib resulted in a slightly higher level of survivin as compared to sorafenib alone. *, p < 0.05, **, p < 0.01 and ***, p < 0.001 using one-way anova/Bonferroni post-hoc test.

To identify a synergistic combination that would permit dose reduction of sorafenib, we opted to use YM155 at the lower 2 mg/kg dose. In the same way, we intentionally deployed sorafenib at a sub-optimal dose of 10 mg/kg, drawing from a previous study carried out on RCC xenograft-bearing SCID mice that higher doses (20 mg/kg, 40 mg/kg) of sorafenib effectively reduced tumour weights by more than 50% of control tumour weights [31]. Disappointingly, xenograft-bearing animals that were treated with this combination (YM155 2 mg/kg, ip, twice daily, 15 days and sorafenib 10 mg/kg, oral, once daily, 15 days) failed to show significant reduction in tumour size or weight. As seen from Fig 9, the combination was more effective than YM155 but no better than sorafenib alone. Interestingly, survivin levels were suppressed in tumours treated with the YM155-sorafenib combination.

Taken together, we have shown that YM155 synergized with sorafenib to enhance cell killing of RCC786.0 cells *in vitro* and that YM155 at 2 mg/kg (ip, 2x daily, x 15 days) suppressed tumour growth in a patient-derived murine RCC xenograft. However, a combination of sub-optimal doses of sorafenib and YM155 failed to demonstrate the desired synergy that would permit dose reduction of sorafenib.

## Discussion

RCC is a highly chemoresistant and radioresistant malignancy with limited treatment options and poor prognosis. Poor outcomes are exacerbated by the presence of a defective *VHL* tumour suppressor gene which is frequently found in RCC tumours. Using a pair of isogenic clear cell RCC786.0 cells (786.0/EV and 786.0/HA.VHL) which differ in VHL status, we found higher survivin levels in VHL-null 786.0/EV than in its VHL positive derivative. A VHL-null patient-derived clear cell RCC cell line (NCC010) also expressed higher survivin levels. The relationship between VHL and survivin may be explained in part by the elevation of survivin in hypoxic conditions. Hypoxia activates hypoxia-inducing factors (HIFs) which upregulate genes that promote cell/tumour proliferation. HIFs are also activated when the *VHL* gene is mutated or defective and this could be one of the factors leading to high survivin levels in VHL null cells. However, silencing survivin in RCC cells, regardless of their VHL status, predictably suppressed cell viability via apoptosis.

Similar to the effect of gene silencing of survivin, the pharmacological inhibition of survivin by YM155, the most widely investigated survivin suppressant, diminished survivability of a varied panel of RCC cells comprising different histological subtypes (clear cell and papillary RCCs) and contrasting VHL status (VHL- null, VHL positive). YM155 consistently demonstrated nanomolar growth inhibitory potencies on all cell lines, was equipotent in suppressing survivin in cells with or without functional VHL and induced apoptotic cell death. YM155 was reported to specifically repress survivin promoter activity by intercepting the binding of transcription factors ILF3/NF110 and SP1 to the promoter, hence blocking transcriptional activity of the survivin gene [18,19]. However, there is growing evidence that suppression of survivin is not the sole and main effect of YM155. The chemical structure of YM155 suggests that it may intercalate DNA, and hence may kill cells primarily by inducing DNA damage [58]. YM155 also intercepts several oncogenic proteins and signaling pathways which could lead to the suppression of survivin [21,22,23,24,25]. Our results reinforce this notion that other factors or mechanism(s) are involved in in the potent growth inhibitory effects of YM155. Thus we showed that suppressing or increasing survivin expression did not abolish or enhance the potency of YM155 on RCC cell lines. A transcriptome analysis of YM155-treated RCC786.0 cells identified up to 200 genes that were differentially expressed in the presence of YM155, the majority of which were associated with the p53 connection pathway and would thus affect survivability, cellular proliferation and cell cycle control. Of these genes, we followed up on four members (tumour suppressors *FOXO1* and *CYLD*; tumour promoters *ID1* and *BIRC5*) that were involved in RCC pathology [5,38,41,45,59,60,61]. *BIRC5* encodes survivin expression [48,49,50] and *FOXO1* mediates cell cycle arrest and promotes apoptosis [41,42]. *ID1* inhibits DNA binding and the transcriptional activity of HLH proteins which leads to tumour growth and angiogenesis [62,63,64]. *CYLD* is a deubiquitin enzyme that inhibits NF-κB signaling [65,66]. mRNA and protein analyses confirmed that YM155 downregulated the tumour promoters BIRC5 and ID1 and upregulated the tumour suppressors FOXO1 and CYLD. Future work should explore the expression levels of these oncogenic proteins before and after YM155 treatment in other clear cell RCC cell lines. If changes are observed, this would provide corroboratory evidence of their involvement in the mode of action of YM155 in clear cell RCC. These investigations which were limited to a random selection of differentially expressed genes relevant to RCC, nonetheless provide useful clues to the broad mechanistic reach of YM155 on signalling pathways within the cell.

In a recent study, YM155 was shown to significantly improve the anticancer activity of temsirolimus in a panel of RCC cell lines *in vitro* and xenograft models *in vivo*. The efficacy of the combination was attributed to strong depletion of survivin by both YM155 and temsirolimus. Sorafenib is as widely used as temsirolimus in RCC and is also known to suppress survivin expression in RCC [31,67]. Thus, we queried if a combination of YM155 and sorafenib would also be clinically advantageous. More important, we were curious if this combination would permit dose reduction of sorafenib which is poorly tolerated at its therapeutic dose. A combination of YM155 and sorafenib at ½ x IC_50_ concentrations, was indeed synergistc when evaluated for growth inhibition in RCC786.0 cells. To translate these findings to the *in vivo* animal model, we confirmed for the first time, the anti-tumour efficacy of YM155 on a murine xenograft model derived from a nephrectomised patient with clear cell RCC. This investigation permitted us to select a suitable dose of YM155 (2 mg/kg) for the combination. A suboptimal dose of sorafenib was likewise selected based on an earlier study. Disappointingly we failed to demonstrate synergy for this combination which was only equivalent to sorafenib given alone in terms of antitumour activity. In retrospect, several aspects of the investigation could have contributed to the outcome. Notably, the doses used in the *in vivo* combination were not optimized and the delivery of YM155 by intraperitoneal injection, in contrast to continuous intravenous infusion which is commonly employed by others, may have compromised its efficacy. It would also be of interest to determine the expression levels of FOXO1, CYLD and ID1 in the patient derived RCC xenograft as there are indications that these proteins are targeted by YM155. These aspects will be addressed in follow-up investigations.

Taken together, our investigations reiterate the critical role of survivin for the growth and proliferation of patient-derived and immortalized RCC cell lines. The small molecule survivin suppressant YM155 potently inhibited survivability of RCC *in vitro* and effectively reduced tumour size in a patient derived RCC xenograft model in mice. Its efficacy as an antitumour agent in RCC is however not exclusively due to inhibition of survivin. A combination of YM155 and sorafenib was synergistic at suboptimal growth inhibitory concentrations in RCC786.0 cells but we could not demonstrate synergy *in vivo* on a patient derived RCC xenograft model.

## Supporting information

S1 Fig Effects of silencing IGF1-R and survivin. Silencing IGF1-R led to a decrease in the expression of survivin. Silencing survivin did not affect the expression of IGF1-R. 100nM of IGF1-R or survivin specific siRNAs was used to effectively induce IGF1-R or survivin gene silencing respectively in RCC786.0/EV cells, RCC786.0/VHL.HA and NCC010 within 48 h following siRNA transfection.
(TIF)

S2 Fig The effects of YM155 on RCC cells. (B) The loading controls of various RCC cell lines treated with increasing concentrations (20 nM– 80 nM) of YM155 for 48 h.
(TIF)

S1 Table siRNA duplexes were supplied by Qiagen (Hilden, Germany). (TIF)

S2 Table miRNA expression vectors produced by the BLOCK-iT^™^ Pol II miR RNAi expression vector kit (Invitrogen, Waltham, MA, USA). (TIF)

S3 Table Primer sequences for sequencing analysis. (TIF)

S4 Table Taqman probe (Applied Biosystems, Waltham, MA, USA) sequences for qRT-PCR analysis. (TIF)

## Abbreviations

CI: Combination Index
CYLD: Cylindromatosis
DMEM: Dulbecco's Modified Eagle Medium
DMSO: Dimethyl sulfoxide
FOXO1: Forkhead box protein O1
HIF: Hypoxia-induced factor
IAP: Inhibitor of apoptosis
ID1: Inhibitor of DNA binding 1
NF-κB: Nuclear factor kappa-light-chain-enhancer of activated B cells
SCID: Severe combined immunodeficiency
tGFP: Turbo green fluorescent protein
YM155: Sepantronium bromide
