# Supplementary figures and images for: Action of YM155 on clear cell renal cell carcinoma does not depend on survivin expression levels
Source: PLoS One. 2017 Jun 5;12(6):e0178168. doi: 10.1371/journal.pone.0178168 (PMC5459331; doi:10.1371/journal.pone.0178168)

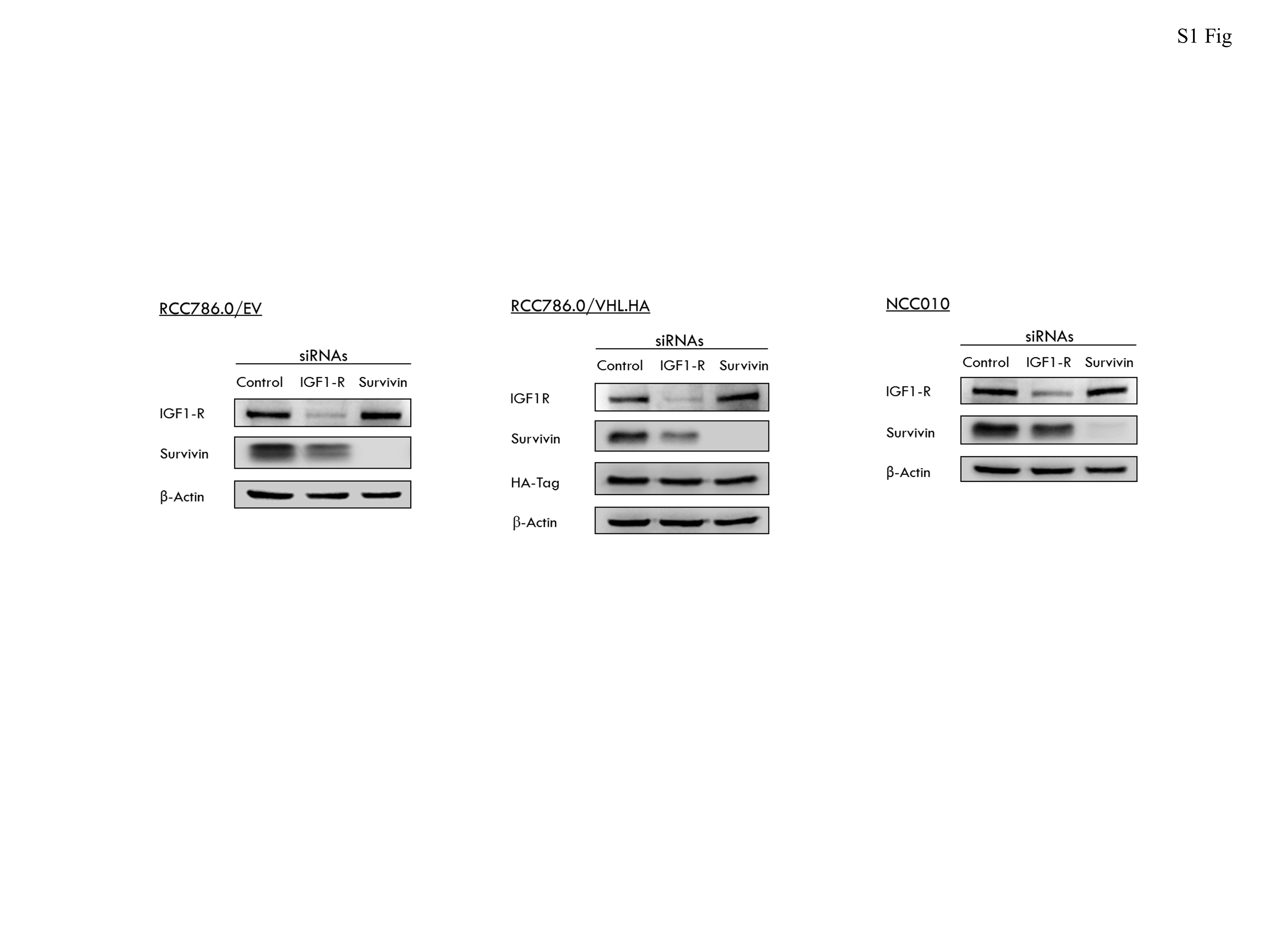

Supplement: S1 Fig — Silencing IGF1-R led to a decrease in the expression of survivin. Silencing survivin did not affect the expression of IGF1-R. 100nM of IGF1-R or survivin specific siRNAs was used to effectively induce IGF1-R or survivin gene silencing respectively in RCC786.0/EV cells, RCC786.0/VHL.HA and NCC010 within 48 h following siRNA transfection. (TIF) [file pone.0178168.s001.TIF]

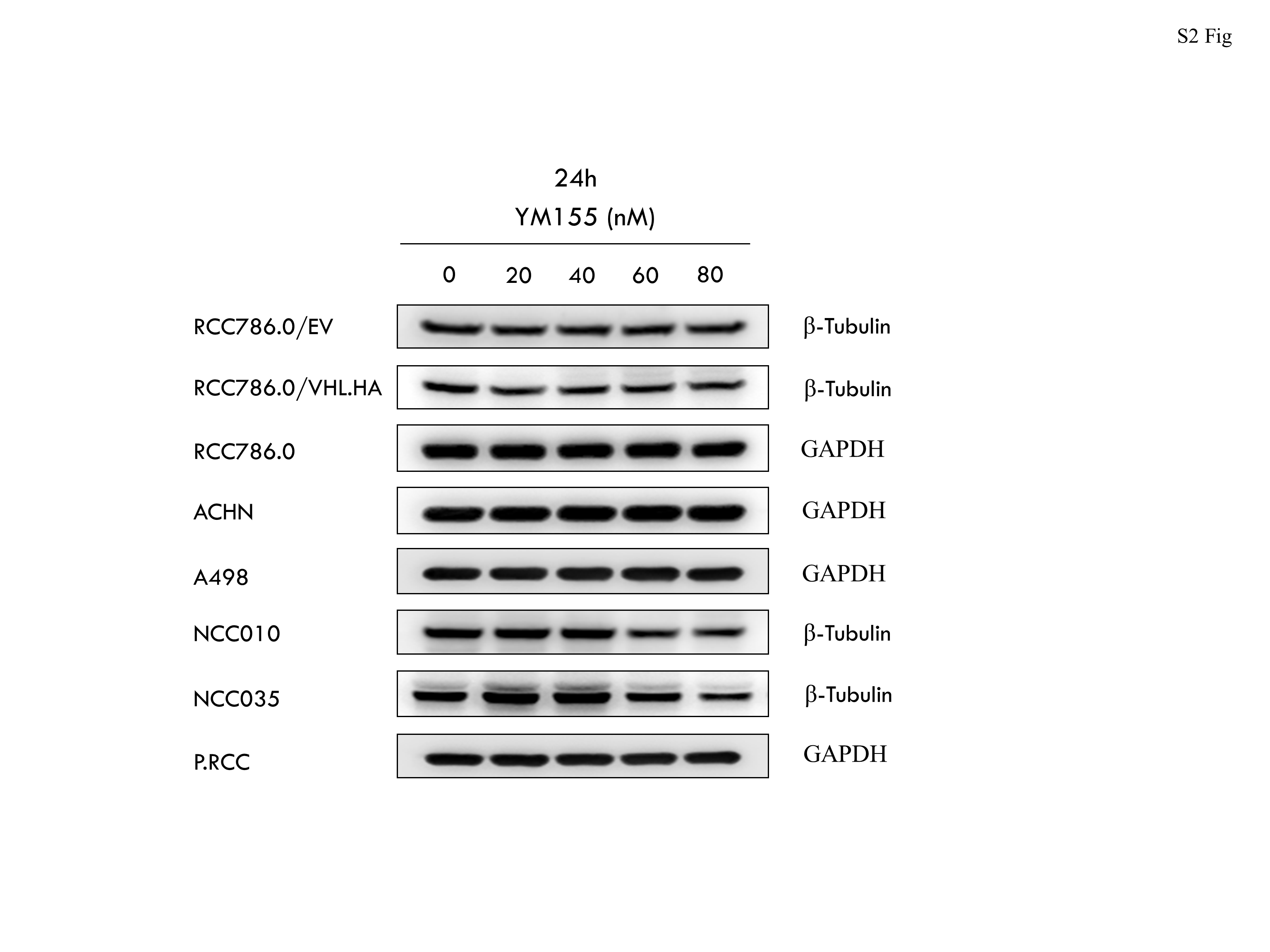

Supplement: S2 Fig — (B) The loading controls of various RCC cell lines treated with increasing concentrations (20 nM– 80 nM) of YM155 for 48 h. (TIF) [file pone.0178168.s002.TIF]

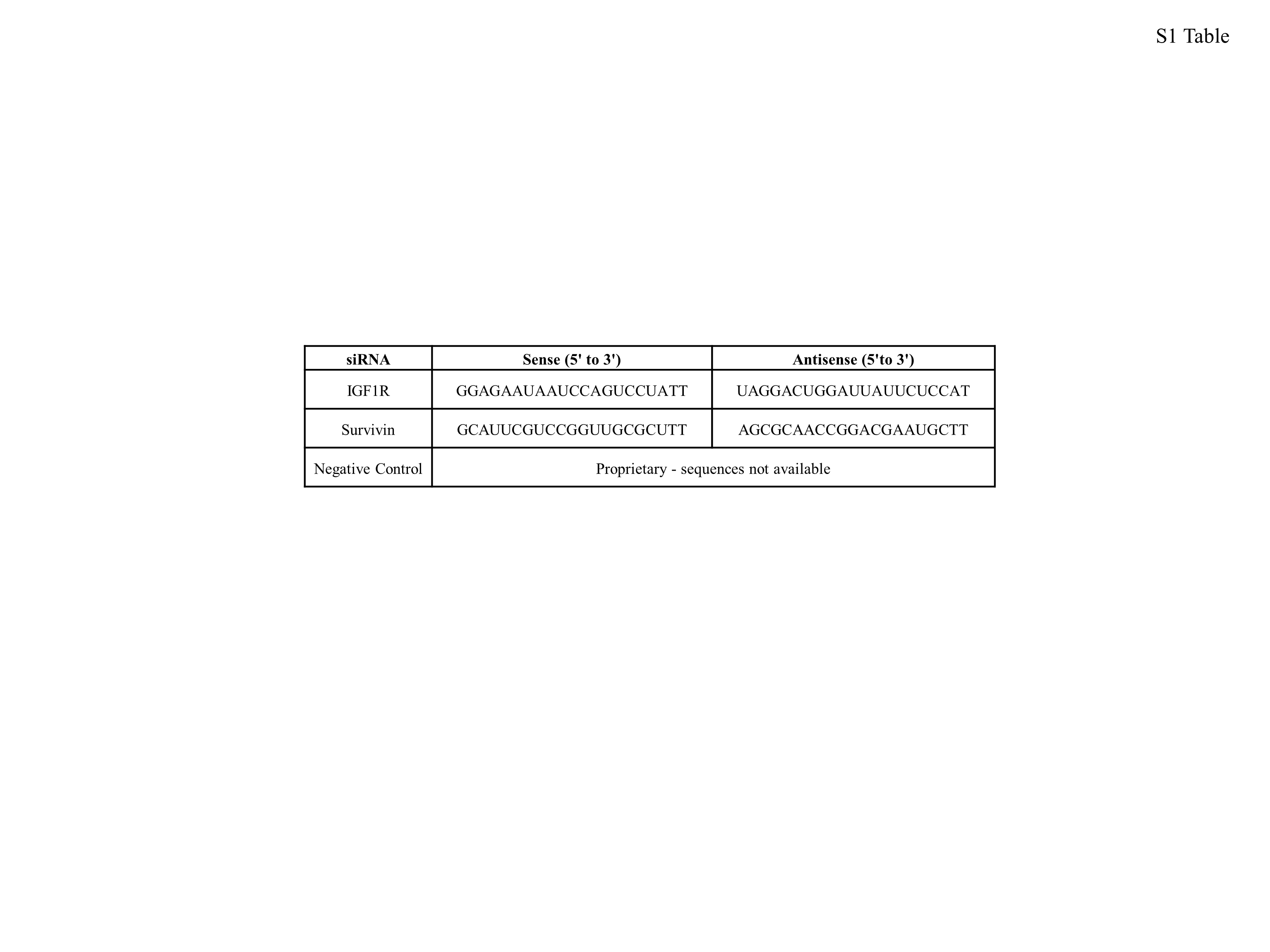

Supplement: S1 Table — (TIF) [file pone.0178168.s003.TIF]

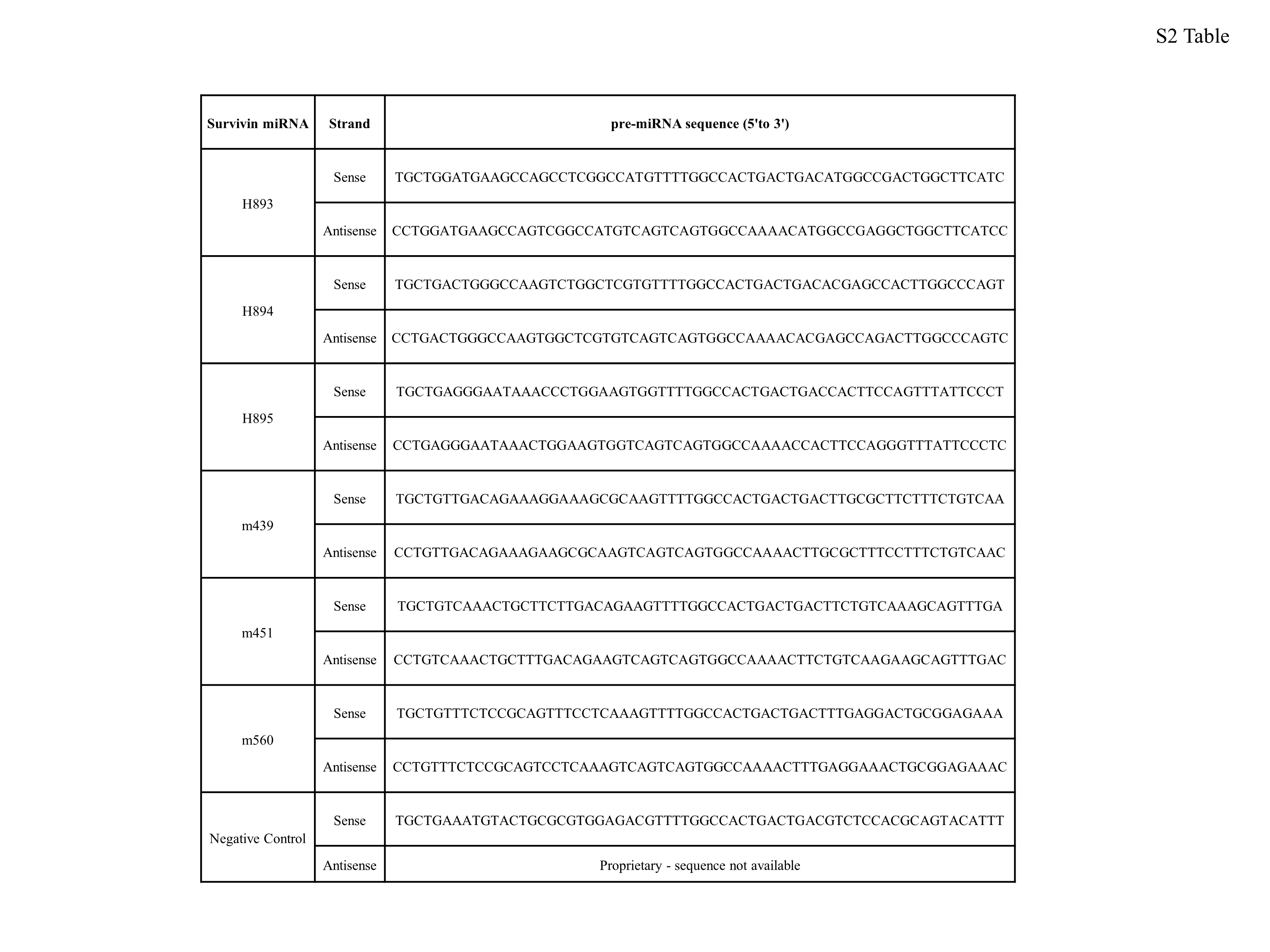

Supplement: S2 Table — (TIF) [file pone.0178168.s004.TIF]

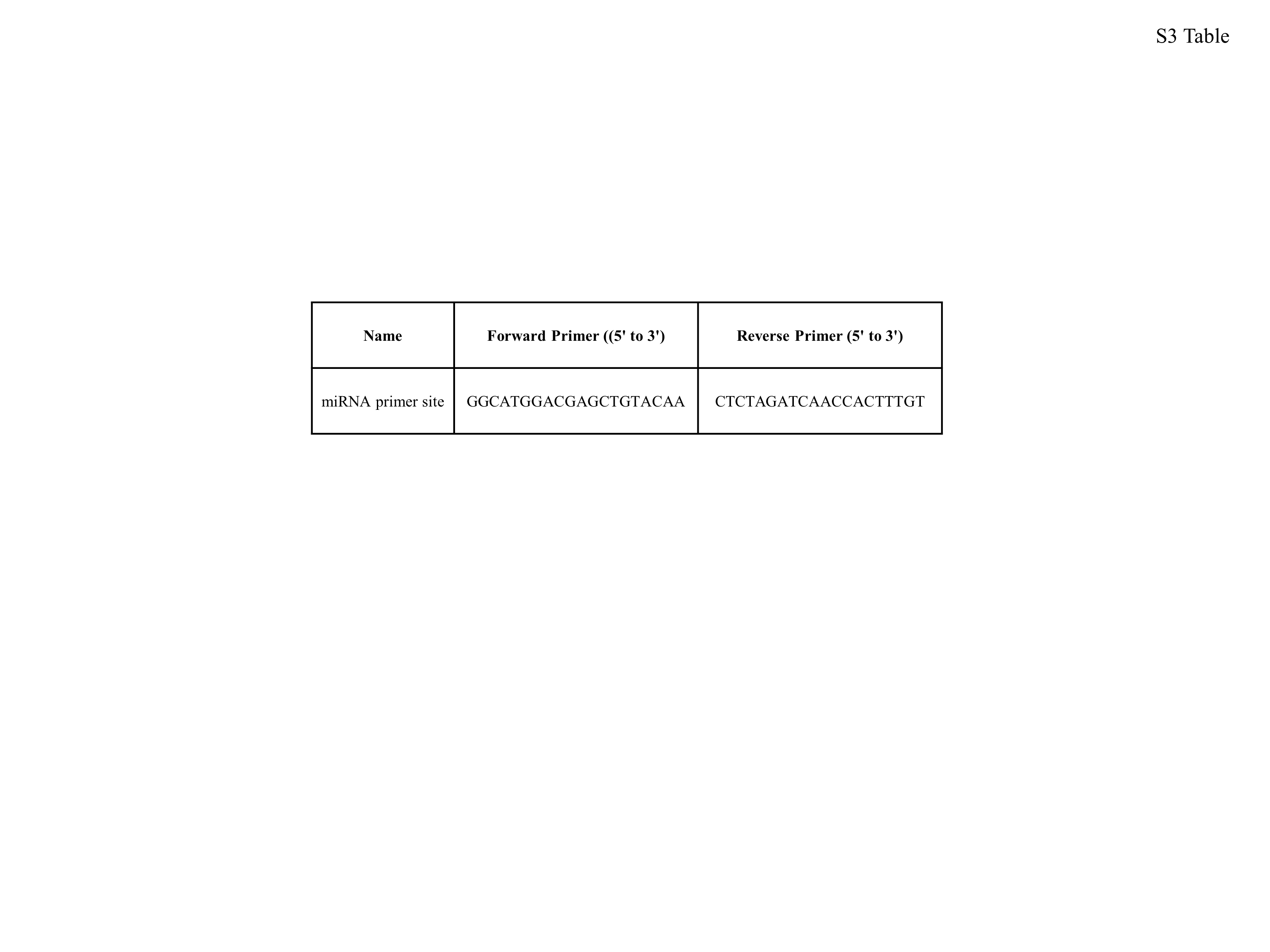

Supplement: S3 Table — (TIF) [file pone.0178168.s005.TIF]

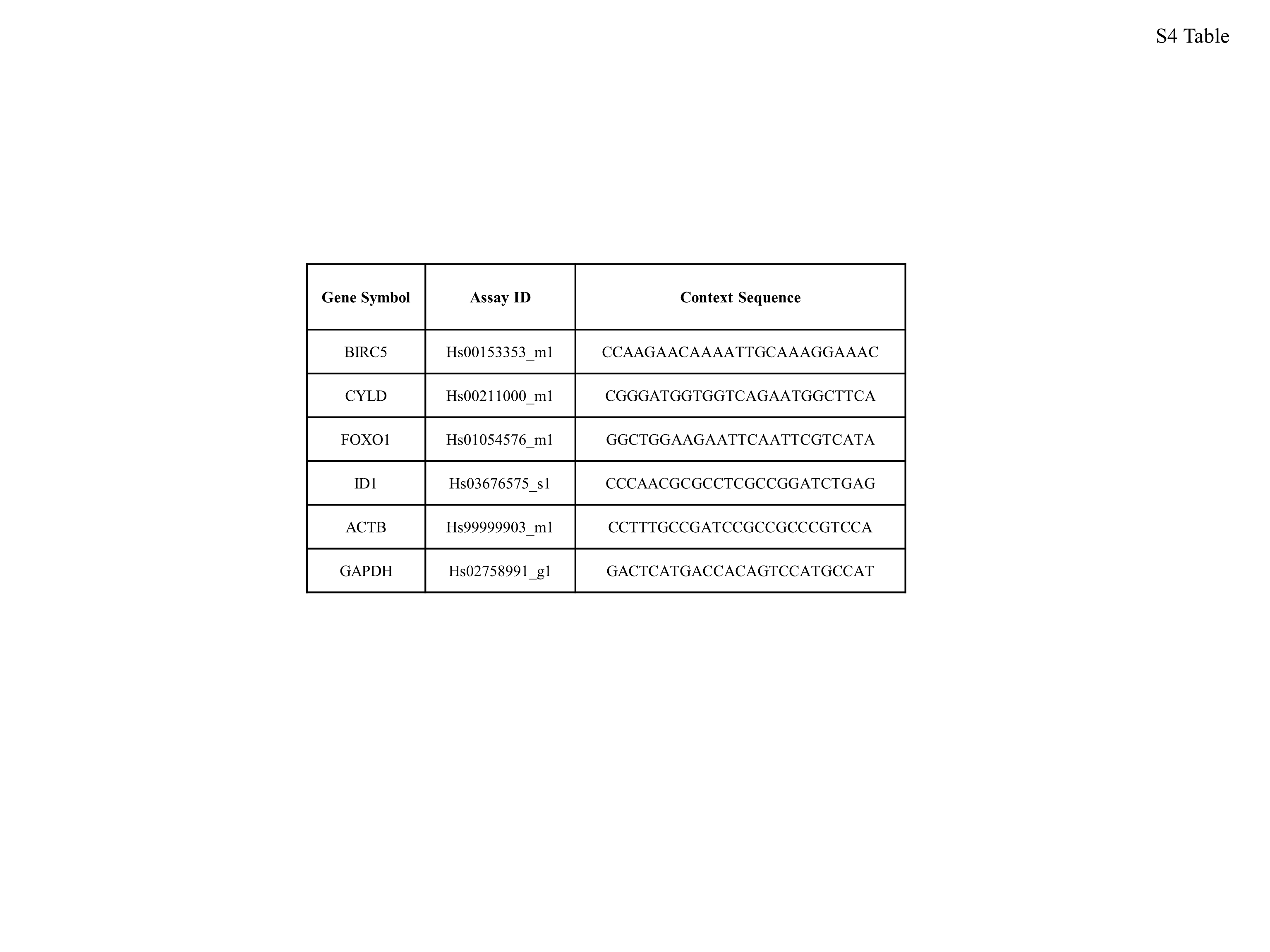

Supplement: S4 Table — (TIF) [file pone.0178168.s006.TIF]
